# Head-to-head comparison of ^99m^Tc-PSMA and ^99m^Tc-MDP SPECT/CT in diagnosing prostate cancer bone metastasis: a prospective, comparative imaging trial

**DOI:** 10.1038/s41598-022-20280-x

**Published:** 2022-09-26

**Authors:** Yu Zhang, Zhiyi Lin, Tao Li, Yongbao Wei, Mingdian Yu, Liefu Ye, Yuqing Cai, Shengping Yang, Yanmin Zhang, Yuanying Shi, Wenxin Chen

**Affiliations:** 1 Department of Nuclear Medicine, Shengli Clinical Medical College of Fujian Medical University, Fujian Provincial Hospital, No. 134. Dongjie Street, Fuzhou, 350001 China; 2 Department of Urology, Shengli Clinical Medical College of Fujian Medical University, Fujian Provincial Hospital, Fuzhou, 350001 China; 3 Department of Medicaledical Department, Shengli Clinical Medical College of Fujian Medical University, Fujian Provincial Hospital, Fuzhou, 350001 China

**Keywords:** Cancer imaging, Prostate, Prostate cancer, Prostate cancer

## Abstract

The most common site of metastasis of prostate cancer (PCa) is bone. Skeletal-related events can increase the risk of death in patients with PCa by 28%. Due to the low detection rate of lesions in patients with low prostate-specific antigen (PSA) levels, the value of ^99m^Tc methylene diphosphonate (^99m^Tc-MDP) bone scintigraphy is limited. Prostate-specific membrane antigen (PSMA) is a small molecular probe that can efficiently and specifically detect PCa lesions. This prospective study aimed to evaluate the difference between ^99m^Tc-PSMA single-photon emission computed tomography (SPECT)/CT and ^99m^Tc-MDP SPECT/CT in the detection of bone metastasis in PCa. A total of 74 men with pathologically confirmed PCa from October 2019 to November 2021 were prospectively enrolled in this study. The median age was 70 (range, 55–87) years. All patients underwent both ^99m^Tc-PSMA SPECT/CT and ^99m^Tc-MDP SPECT/CT at an average interval of 12.1 (range, 1–14) days. The detected imaging-positive bone lesions were scored as “typical metastasis” or “equivocal metastasis” by a standard reporting schema. Subsequent therapy modality details were observed through follow-up. Twenty-five of the 74 patients were diagnosed with bone metastases. ^99m^Tc-PSMA SPECT/CT and ^99m^Tc-MDP SPECT/CT detected 20 and 18 bone metastases, with sensitivities of 80.0% (20/25) and 72.0% (18/25), specificities of 100.0% (49/49) and 81.3% (40/49), and AUCs of 88.0% and 84.9%, respectively. There was a significant difference in the AUC between the two imaging methods (*P* < 0.001). In an analysis of the number of bone metastasis lesions, the proportion of “typical metastasis” versus “equivocal metastasis” detected by the two imaging methods was 26.3:1 (PSMA) and 2.9:1 (MDP), and the difference was statistically significant (*P* = 0.005). There was a significant difference in the detection of bone metastatic lesions by ^99m^Tc-PSMA and ^99m^Tc-MDP when the maximum diameter of the lesions was ≤ 0.6 cm (*P* < 0.05). The optimal cut-off value for PSA was 2.635 ng/mL (PSMA) and 15.275 ng/mL (MDP). ^99m^Tc-PSMA SPECT/CT led to a change in management to a more individualized therapy modality for 11 of 74 men (14.9%). ^99m^Tc-PSMA SPECT/CT was superior to ^99m^Tc-MDP SPECT/CT in the detection of bone metastases in PCa, especially for small lesions and in patients with low PSA levels, and demonstrated an additional benefit of providing information on extraskeletal metastases. With regard to therapy, ^99m^Tc-PSMA scans might have utility in improving the subsequent therapy modality.

## Introduction

Prostate cancer (PCa) is the most common cancer of the urinary system in older men and the sixth leading cause of cancer-related death^1^. Bone is the most common site of distant organ metastasis in PCa, with 8% to 35% of patients already having distant metastasis at diagnosis^2^. Skeletal-related events (SREs) increase the risk of death by 28% in patients with PCa^3^. ^99m^Tc methylene diphosphonate (^99m^Tc-MDP) bone scintigraphy (BS) is a sensitive and relatively economical method for detecting bone metastases in PCa. However, its specificity is limited, and many benign bone diseases may show false positive results on BS^4^.

In recent years, prostate cancer-specific membrane antigen (PSMA) probe imaging technology has undergone rapid development. PSMA is a type II transmembrane glycoprotein (100 kDa) expressed in prostate epithelial cells and demonstrates glutamic carboxypeptidase (GCP-II) and folic acid hydrolase activity. PSMA is highly overexpressed on the surface of 90% of PCa cells, 1000 times more than in normal tissue. The expression of PSMA is further increased in high-risk PCa, metastatic PCa and castration-resistant prostate cancer (CRPC)^5^. Related preclinical studies have shown that ^99m^Tc-labelled PSMA molecular probe (^99m^Tc-HYNIC-Glu-Urea-A, hereinafter referred to as ^99m^Tc-PSMA) single–photon emission computed tomography (SPECT)/CT can clearly display PCa lesions with positive PSMA expression, and the imaging agent is quickly cleared from the blood, mainly through kidney metabolism. Additionally, there is only a small amount of radiation uptake in the intestinal tract and no significant radiation uptake in other major organs^6^. Our study aimed to investigate the difference between ^99m^Tc-PSMA SPECT/CT and ^99m^Tc-MDP SPECT/CT in the detection of bone metastasis in PCa.

## Materials and methods

This study was approved by the ethics committee of Fujian Provincial Hospital (reference number, K2019-10-017), and all methods were carried out following relevant guidelines and regulations. This study was carried out in compliance with the Declaration of Helsinki. Informed consent was obtained from all participants and/or their legal guardians.

### Sample size calculation

We conducted a prospective observational study to analyse the difference between ^99m^Tc-PSMA SPECT/CT and ^99m^Tc-MDP SPECT/CT in the detection of bone metastasis in PCa. In this study, the sensitivity and specificity of ^99m^Tc-PSMA and ^99m^Tc-MDP scans were assumed to be greater than 50% (H_0_ = 50%). Referring to similar published literature on ^68^ Ga-PSMA and ^99m^Tc-MDP scans^7,8^, an H1 = 80% was assumed. PASS 11 software (power analysis and sample size, NCSS, LLC) was used to estimate the required sample size. Assuming α = 0.05 (unilateral), β = 0.1, and a 1:1 ratio between groups, the calculations indicated that at least 46 patients needed to be included in the study. Ultimately, 74 individuals were enrolled in the study.

### Patient selection

A total of 74 men were enrolled in this study from October 2019 to November 2021. The inclusion criteria were as follows: (1) PCa confirmed by surgical or puncture histopathology; (2) completion of initial treatment [such as radical prostatectomy (RP), external-beam radiotherapy (EBRT), endocrine therapy, etc.]; (3) biochemical recurrence (defined as: (i) a PSA level ≥ 0.2 μg/L on two consecutive measurements after RP; (ii) after EBRT, any PSA increase > 2 ng/mL higher than the PSA nadir value, regardless of the serum concentration of the nadir)^9^; and (4) complete medical records, control data and clinical follow-up results. The exclusion criteria were as follows: (1) the presence of sever syndromes that were difficult to manage; (2) active or upcoming participation in other clinical drug trials; (3) lack of regular review or follow-up results; and (4) inability to obtain relevant contrast imaging and clinical data. All eligible patients underwent both ^99m^Tc-PSMA SPECT/CT and ^99m^Tc-MDP SPECT/CT at an average interval of 12.1 days (1–14 days). None of the patients received any antineoplastic therapy between the two scans. The characteristics of the patients are given in Table 1.

**Table 1:** Patient characteristics.

Patient characteristic	Value
**No. of patients**	74
Primary staging, n (%)	19 (25.7%)
BR, n (%)	19 (25.7%)
Restaging, n (%)	36 (48.6%)
Age (years)*	70 (55–87)
Serum PSA (ng/ml)*	15.56 (0.01–906.20)
0.00 ~ 9.99, n (%)	50 (67.5%)
10.00 ~ 20.00, n (%)	13 (17.5%)
> 20.00, n (%)	11 (15.0%)
**Gleason score**	
< 7 (low risk), n (%)	8 (10.8%)
= 7 (intermediate risk), n (%)	13 (17.6%)
> 7 (high risk), n (%)	53 (71.6%)
**Prostatectomy**	
Yes, n (%)	49
No, n (%)	25

*BR* biochemical recurrence. * data are expressed as the median (range).

### Radiopharmaceuticals

A PSMA lyophilized kit was provided by Shanghai Engineering Research Center of Molecular Imaging Probes. Before each use, a bottle of lyophilized reagent was selected, and after 5 min, 4 mL 0.9% NaCl solution was added to dissolve the reagent, followed by approximately 5 mL 3.7–4.44 GBq ^99m^TcO^-^_4_ solution. The solution was then mixed well and heated in a 100 °C water bath for 10 min. The radiochemical purity (RCP) of the ^99m^Tc-PSMA was assessed by analytical high-performance liquid chromatography (HPLC) on an Agilent 1200 system^6^. The ^99m^Tc-PSMA was discarded if the RCP was lower than 95%. The ^99m^Tc-MDP was provided by Guangdong CI Pharmaceutical Co., LTD. Fuzhou Branch. Quality control (QC) of ^99m^Tc-MDP was carried out by the manufacturer.

### Imaging protocol

For the ^99m^Tc-PSMA scan, all patients were injected intravenously with a dose of 0.74 GBq (20 mCi) ^99m^Tc-PSMA. Whole-body planar imaging and regional (neck-pelvic) SPECT/CT were performed 2 h after injection on a Discovery NM/CT 670Pro (GE, USA) with low energy high resolution collimators. The image acquisition protocol was as follows: (1) Planar imaging: peak energy 140 keV (^99m^Tc) and scan velocity 15 cm/min in a 1025 × 256 matrix. (2) Regional SPECT/CT: camera matrix size 128 × 128, zoom 1.0, rotation 360°, and 30 s/frame for a total of 60 frames. For CT, low-dose CT (130 keV; 60 mAs) was used.

For the ^99m^Tc-MDP scan, a dose of 0.74 GBq (20 mCi) ^99m^Tc-MDP was injected intravenously, and imaging was performed following a delay of 3 to 5 h. The imaging instrument and acquisition protocol were the same as those of the ^99m^Tc-PSMA scan.

### Image analysis

**Figure 1:**
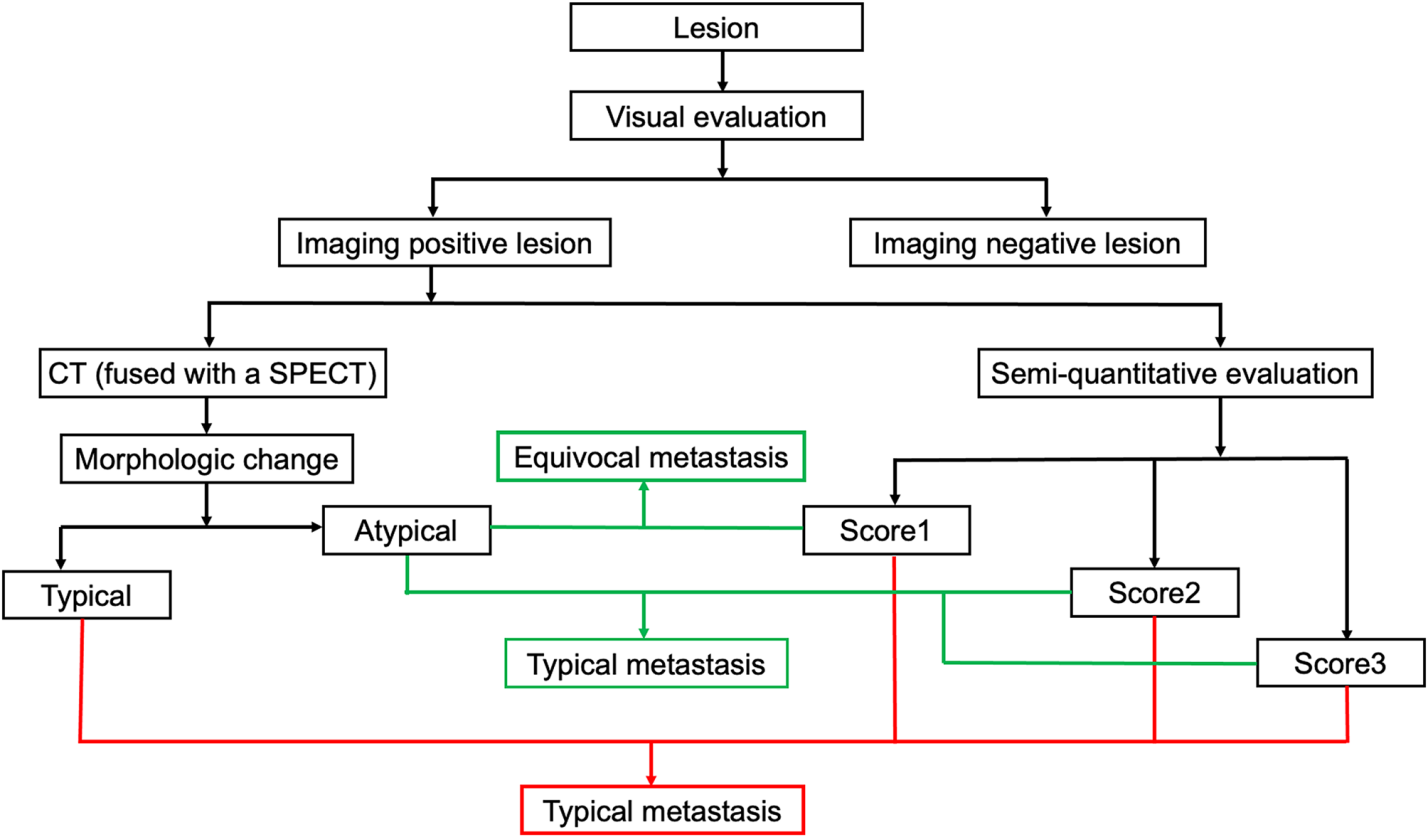
Image analysis flowchart.

Image processing was performed on workstations (Xeleris, General Electric, Waukesha, WI). All images were anonymized and analysed by 3 senior nuclear medicine physicians. On SPECT/CT, areas with higher imaging agent uptake than normal tissue after excluding physiological uptake and traumatic fracture were considered “imaging positive bone lesions”. Areas with abnormal SPECT/CT findings but no imaging agent uptake on the corresponding site of SPECT were considered negative lesions. The flow diagram is shown in Fig. 1.

#### Interpretation of the degree of uptake of the positive lesion imaging agent by semiquantitative evaluation

(1) For the ^99m^Tc-PSMA scan, the region of interest (ROI) of the lesions was delineated on whole-body plane imaging, and a mirror ROI was delineated on the liver, avoiding the gallbladder to the greatest extent possible. Then, the ratio of focus to liver (F/L) was calculated; if F/L > 1, the lesion was regarded as having high uptake (score: 3); if F/L = 1, it was regarded as having moderate uptake (score: 2); and if F/L < 1, it was regarded as having mild uptake (score: 1). (2) For the ^99m^Tc-MDP scan, the ROI of the lesions was delineated on whole-body plane imaging, and the mirror ROI was delineated on the contralateral part (if the lesion was located in the spine, the adjacent vertebrae were delineated), and the ratio of focus to contralateral (F/CL) was calculated. If F/CL ≥ 2, lesion uptake was regarded as high (score 3); if F/L was 1.5–2, it was regarded as moderate (score: 2); and if F/L was 1–1.5, it was regarded as mild (score: 1).

#### Diagnostic criteria for “typical metastasis” and “equivocal metastasis”

(1) Diagnostic criteria for “typical metastasis” (i) typical metastatic changes (increased bone density and/or destruction of bone structure, with or without surrounding soft tissue mass) on CT (fused with SPECT) of the lesion and an imaging score of 1–3. (ii) lack of typical benign or metastatic changes on CT (fused with SPECT) and an imaging score of 2–3. (2) Diagnostic criteria for “equivocal metastasis”: (iii) no typical benign or metastatic changes on CT (fused with SPECT) and an imaging score of 1.

### Validation criteria for bone metastases

The pathological criteria for bone metastasis are difficult to obtain; thus, the clinical diagnosis method reported in a previous study was used^7^. All patients were followed up for at least 6 months (or until death). Serum PSA was reviewed every 3 months for all patients. Subsequent therapeutic schedule options depended on the patient's condition, including radical prostatectomy, local radiation therapy, chemotherapy, abiraterone, etc. ^99m^Tc-PSMA and ^99m^Tc-MDP imaging were routinely performed every 6 months to describe changes in activity on bone lesions. Future imaging modalities (CT, magnetic resonance (MR), positron emission tomography (PET)/CT, PET/MR, etc.) were selected according to their respective clinical needs and were not bound by a specific protocol. For patients with BR, ^18^F-FDG PET/CT was performed annually. One of these patients was found left supraclavicular fossa lymph node metastases both by ^18^F-FDG PET/CT and ^99m^Tc-PSMA SPECT/CT at follow-up one year later.The material was assessed by the specialists involved in the study (nuclear medicine physicians and urologists) to determine the affected bone regions and overall metastatic status. Patients who met at least two of the following conditions were clinically diagnosed with bone metastasis: (1) two or more imaging scans suggestive of bone metastases; (2) symptoms of bone pain and imaging examination suggesting bone metastasis at the site of bone pain, which was relieved after antitumor therapy; (3) a reduction in size or activity for positive metastases after antitumor therapy on imaging examination; and (4) PSA ≥ 100 ng/mL, suggesting distant metastasis^10^.

### Statistical analysis

Data analysis was performed using SPSS 19.0 software (statistical product and service solutions, Chicago, IL). The sensitivity and specificity of the two imaging methods were calculated, and using receiver operating characteristic (ROC) curve analysis, the area under the ROC curve (AUC) was calculated, compared and analysed between the two methods. The Wilcoxon signed-rank test was used to analyse the difference between the proportion of “typical metastasis” versus “equivocal metastasis” as detected by the two imaging methods. The Wilcoxon rank-sum test was used to analyse the difference in the number of bone metastatic lesions detected by the two imaging methods. Binary logistic regression analysis and ROC curve analysis were used to calculate the predictors and optimal critical values of the positive results of the two imaging methods. *P* < 0.05 was considered statistically significant.

## Results

### Overall results

**Figure 2:**
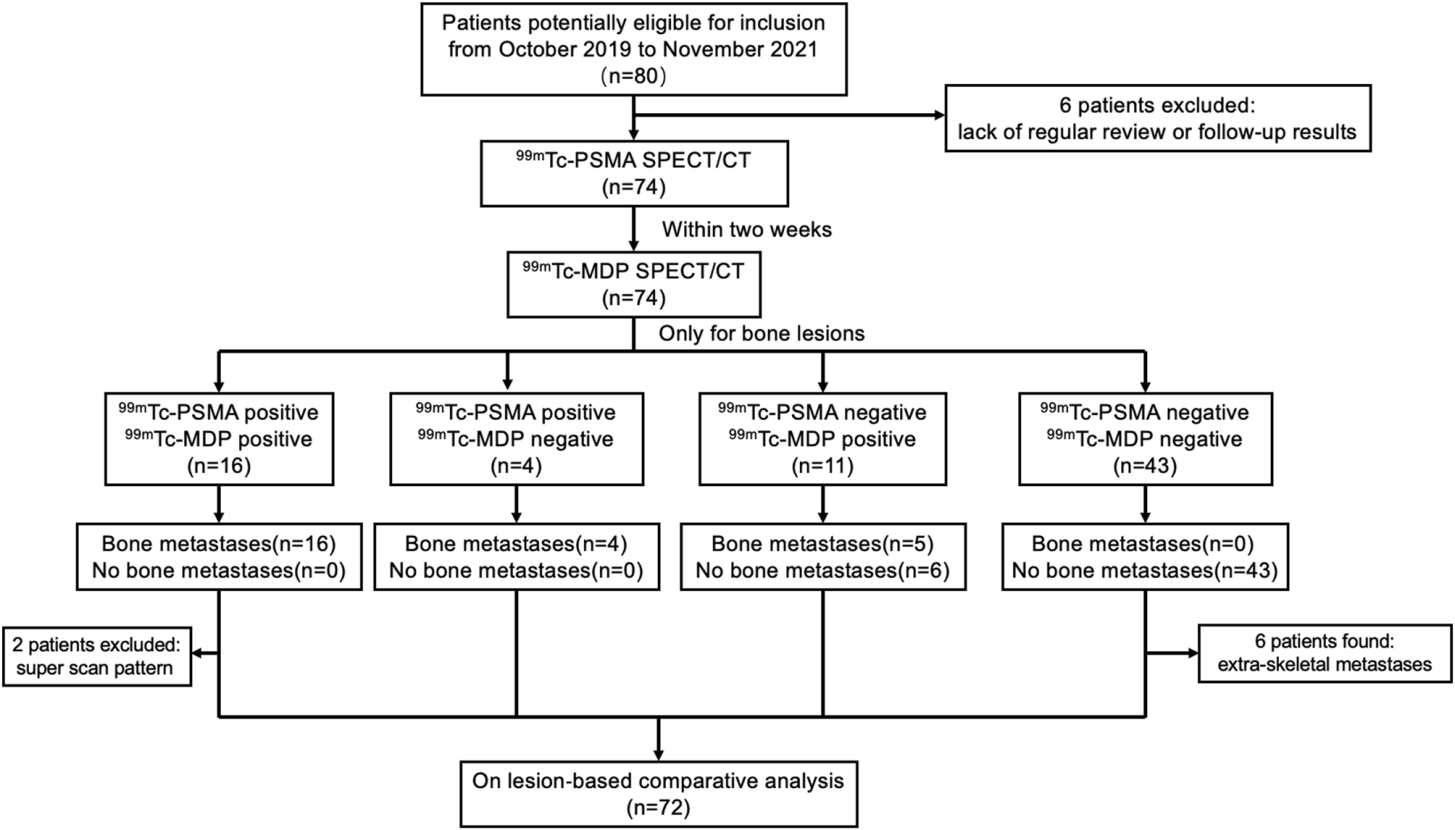
Flowchart of participant selection in the study.

Among the 74 patients, 25 (33.8%) were diagnosed with bone metastasis, and 49 (66.2%) did not have bone metastasis. A flowchart illustrating the participant inclusion procedure in the study is shown in Fig. 2. Among the 74 patients, 2 with systemic diffuse bone metastases (similar to bone super scans) were excluded from the analysis, mainly because the number of bone lesions in these patients was difficult to count. A total of 109 bone lesions were identified by ^99m^Tc-PSMA, of which 105 were confirmed as bone metastases during follow-up. A total of 106 bone lesions were identified by ^99m^Tc-MDP, of which 84 were confirmed as bone metastases during follow-up.

### Sensitivity and specificity analysis

**Figure 3:**
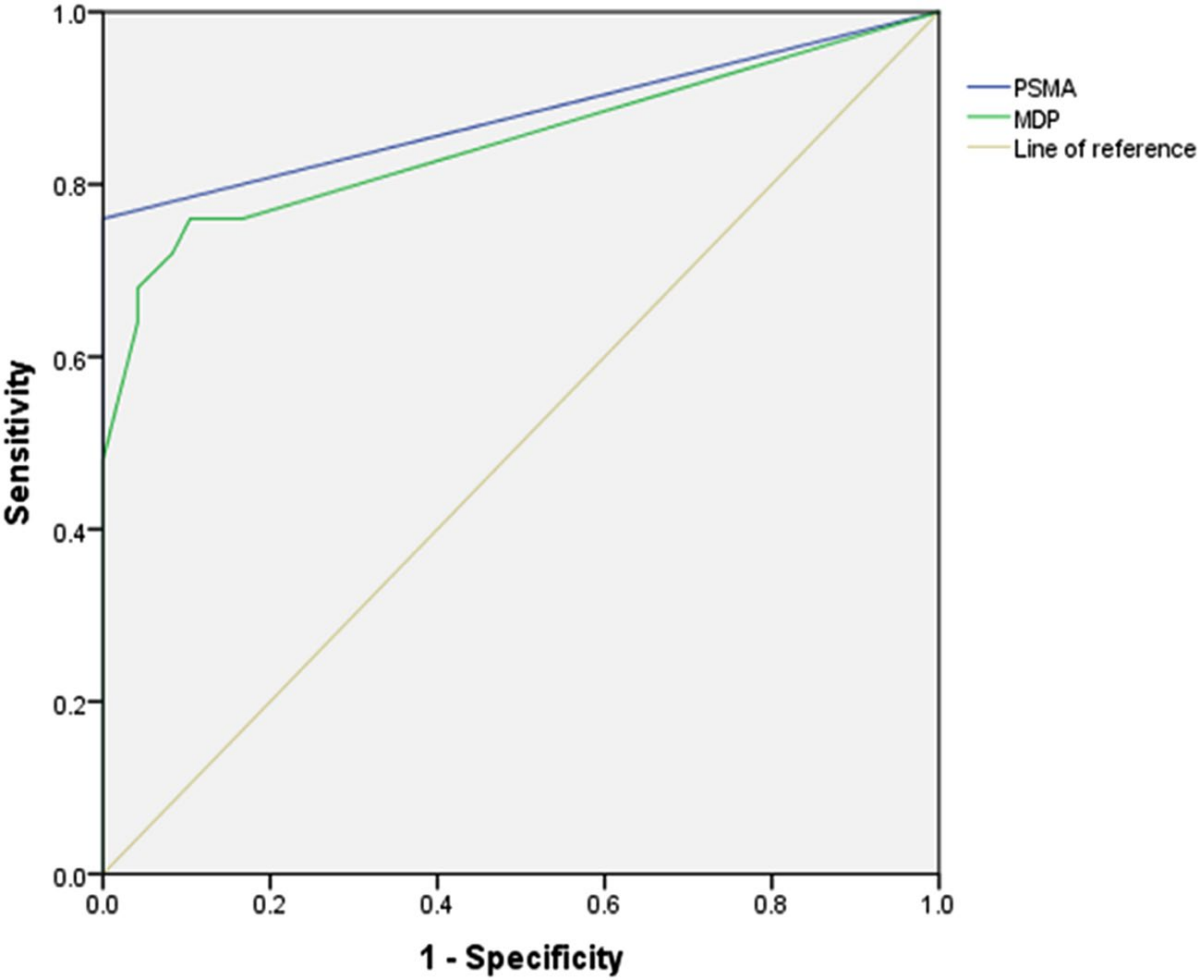
Receiver operating characteristic (ROC) curves for ^99m^Tc-PSMA SPECT/CT and ^99m^Tc-MDP SPECT/CT.

For all 74 patients, the sensitivity of ^99m^Tc-PSMA SPECT/CT and ^99m^Tc-MDP SPECT/CT in the detection of bone metastasis was 80.0% (20/25, *95% CI.* 50.7–92.4%) and 72.0% (18/25, *95% CI.* 50.4–87.1%), respectively, and the specificity was 100.0% (49/49, *95% CI.* 90.8–100.0%) and 81.3% (40/49, 95% *CI.* 66.9–90.1%). ROC curve analysis revealed an accuracy, as measured by AUC, of 88.0% (*95% CI.* 77.7–98.3%) for ^99m^Tc-PSMA and 84.9% (95% *CI.* 73.8–95.9%) for ^99m^Tc-MDP. The difference in AUC between the two methods was statistically significant (*P* < 0.001) (Table 2 and Fig. 3).

**Table 2:** ^99m^Tc-PSMA SPECT/CT and ^99m^Tc-MDP SPECT/CT in the diagnosis of bone metastasis.

Clinical diagnosis	^99m^Tc-PSMA		^99m^Tc-MDP	
	Positive	Negative	Positive	Negative
Positive (n = 25)	20	5	18	7
Negative (n = 49)	0	49	9	40
Total	20	54	27	47
AUC	0.880		0.849	
SE	0.052		0.056	
95% CI	0.777–0.983		0.738–0.959

### Metastasis and equivocality analysis

The proportions of “typical metastasis” versus “equivocal metastasis” detected by the two imaging methods were 26.3:1 (PSMA) and 2.9:1 (MDP), respectively, and the difference was statistically significant (*P* = 0.005) according to the Wilcoxon signed-rank test (Table 3).

**Table 3:** Ratio of equivocal or metastatic lesions.

	Type of imaging positive bone lesion			
	^99m^Tc-PSMA		^99m^Tc-MDP	
	Metastasis	Equivocal	Metastasis	Equivocal
No. of Lesions (n)	105	4	79	27
radio	26.3:1		2.9:1	
*P* value	0.005			

### Analysis of the number of bone metastatic lesions

**Figure 4:**
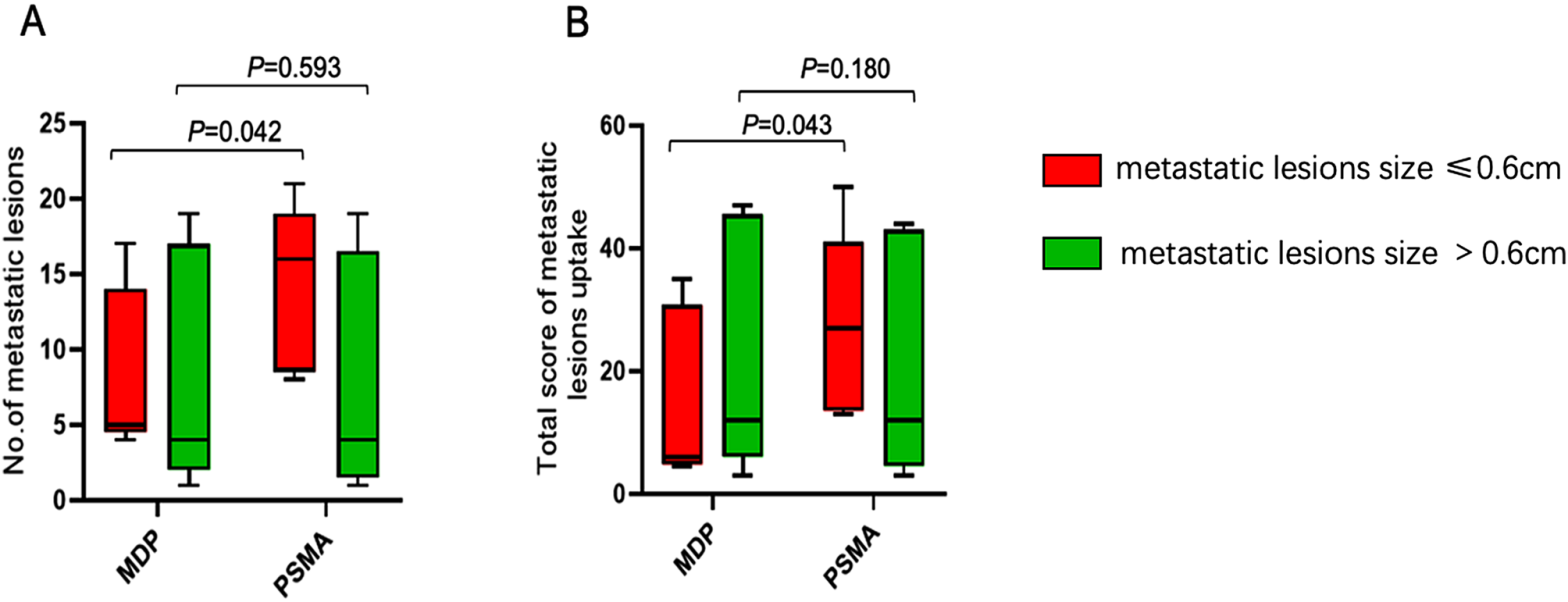
Comparison of metastatic lesions between the two agents. (**A**): Number of metastatic lesions detected by ^99m^Tc-PSMA and ^99m^Tc-MDP. (**B**): Degree of uptake on ^99m^Tc-PSMA and ^99m^Tc-MDP imaging.

The ^99m^Tc-PSMA and ^99m^Tc-MDP scans detected 105 and 84 bone metastases, respectively. In our centre, the spatial resolution of SPECT was 0.6 cm. Thus, according to the maximum diameter of the bone metastatic lesions measured on CT (fused with SPECT), the lesions were divided into two groups: G1 (maximum diameter ≤ 0.6 cm) and G2 (maximum diameter > 0.6 cm). The rank-sum test showed that there was a significant difference in the number of bone metastatic lesions, and the degree of uptake of the imaging agent observed under ^99m^Tc-PSMA was better than that of ^99m^Tc-MDP (both *P* < 0.05) when the maximum diameter of the bone metastatic lesions was ≤ 0.6 cm (Table 4 and Fig. 4).

**Table 4:** Number of clinical bone metastases and degree of imaging agent uptake observed under ^99m^Tc-PSMA and ^99m^Tc-MDP.

	Bone metastatic lesion size groups							
	≤ 0.6 cm				> 0.6 cm			
	PSMA	MDP	*Z* value	*P* value	PSMA	MDP	*Z* value	*P* value
Diameter*	0.50 (0.30, 0.60)	0.50 (0.38, 0.60)	− 1.350	0.177	0.70 (0.70, 0.80)	0.80 (0.70, 0.80)	− 1.515	0.130
Number**	16.00 (8.50, 19.00)	5.00 (4.50, 14.00)	− 2.032	0.042	2.00 (1.50, 14.50)	4.00 (2.00, 17.00)	− 0.535	0.593
Score***	27.00 (13.50, 41.00)	6.00 (4.75, 30.75)	− 2.023	0.043	12.00 (4.50, 43.00)	12.00 (6.00, 45.50)	− 1.342	0.180

*Maximum diameter of bone metastatic lesion expressed in cm.

**Number of bone metastatic lesions.

***Total score of bone metastatic lesion uptake.

### Relationship between PSA level and bone metastases

To exclude the influence of the tumour load of extraskeletal metastases on the PSA level, we selected patients who underwent RP and did not have extraskeletal metastases for inclusion in this cohort. In our study population, 49/74 patients received RP, and 8 of these patients were found to have extraskeletal metastases. Consequently, a total of 41 patients with no extraskeletal metastases after RP were included in this cohort analysis. With the presence of bone metastasis on ^99m^Tc-PSMA (yes = 1, no = 0) as the dependent variable and age, serum PSA level, and the Gleason score as the independent variables, binary logistic regression analysis showed that serum PSA was independently correlated with the presence of bone metastases on ^99m^Tc-PSMA: specifically, for every 1 ng/mL increase in serum PSA level, the detection rate of bone metastases increased by 4.4% (*OR* = 1.044, *P* < 0.001) (Table 5). ROC analysis revealed an optimal PSA cut-off value of 2.635 ng/mL, with an AUC of 0.800 in predicting ^99m^Tc-PSMA scan positivity, a sensitivity of 85.0%, a specificity of 76.7% and Youden’s index of 0.517. In binary logistic regression analysis, a relationship was uncovered between the presence of bone metastases on ^99m^Tc-MDP and PSA level and the Gleason score, but no relationship with age was demonstrated (*P* > 0.05). For every 1 ng/mL increase in serum PSA level, the detection rate of bone metastases increased by 3.8% (*OR* = 1.038, *P* < 0.001) (Table 5). ROC analysis demonstrated an optimal PSA cut-off value of 15.275 ng/mL, with an AUC of 0.740 in predicting ^99m^Tc-MDP scan positivity, a sensitivity of 66.7%, a specificity of 79.5% and Youden's index of 0.462.

**Table 5:** Logistic regression analysis of related factors for the presence of clinical bone metastases on ^99m^Tc-PSMA and ^99m^Tc-MDP.

Imaging method	Independent variable	*OR* value	95% *CI* for OR		*P* value
			Lower	Upper	
^99m^Tc-PSMA	PSA	1.044	1.009	1.080	< 0.001
	Age	1.097	0.977	1.232	0.116
	Gleason score	4.123	1.372	12.393	0.012
^99m^Tc-MDP	PSA	1.038	1.008	1.069	< 0.001
	Age	1.102	0.969	1.254	0.139
	Gleason score	4.072	1.261	13.151	0.003

*CI* confidence interval, *OR* odds ratio.

### Improvement in subsequent therapy modality

**Figure 5:**
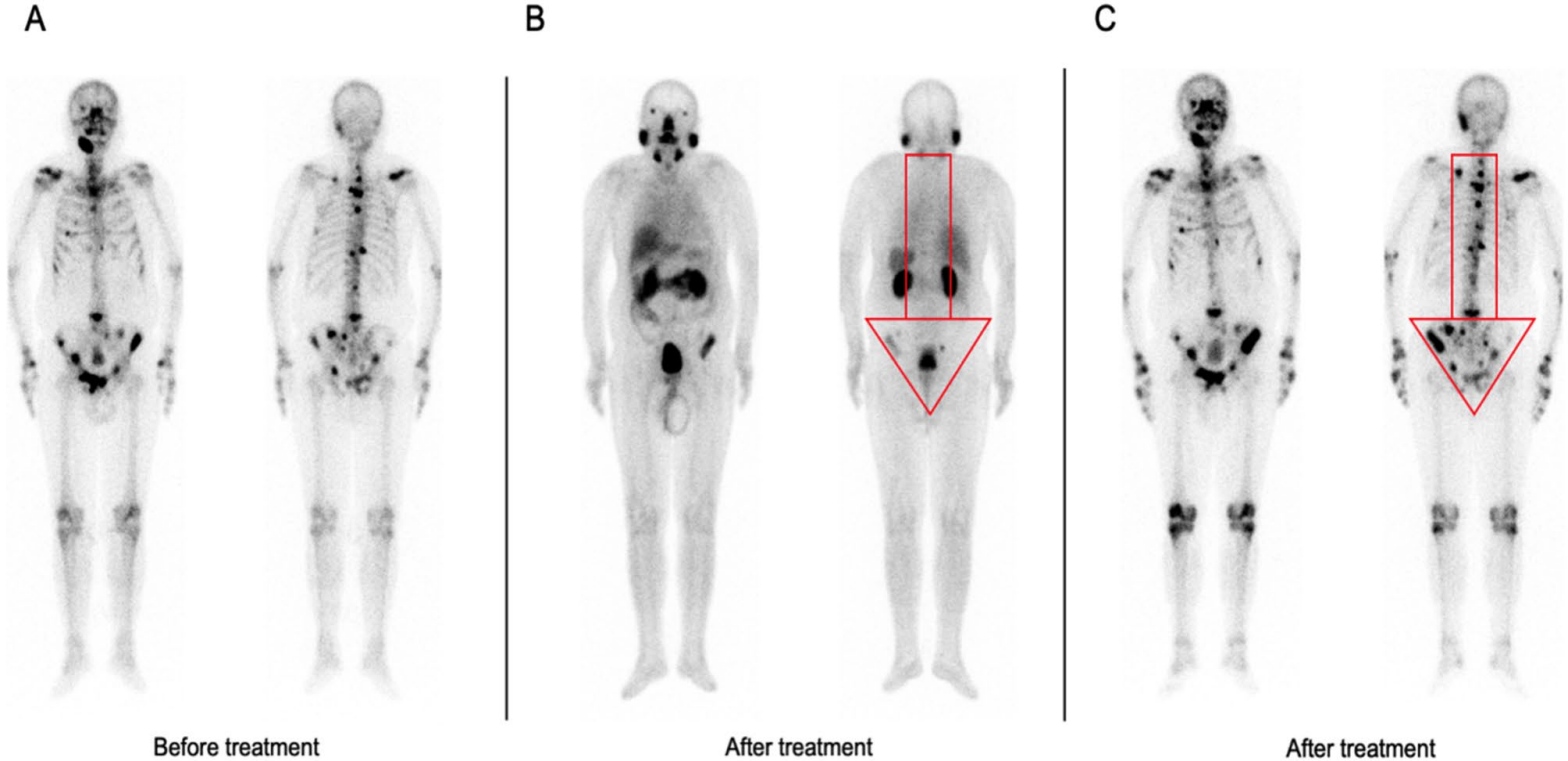
83-year-old patient with a restaged PCa. The Gleason score was 9, and the PSA level was 587.66 ng/mL before treatment. (**A**): Whole-body planar ^99m^Tc-MDP before treatment. (**B**): Whole-body planar ^99m^Tc-PSMA after treatment. (**C**): Whole-body planar ^99m^Tc-MDP after treatment. The PSA level was 8.05 ng/mL after 8 months of androgen deprivation therapy (ADT). There was a high degree of inconsistency between the ^99m^Tc-PSMA scan and ^99m^Tc-MDP scan after treatment.

In 11 of 74 patients (14.9%), ^99m^Tc-PSMA SPECT/CT would most likely have changed the management into a different therapy modality. The most common improvement in therapy modality was a change in the radiotherapy approach (10/11, 90.9%). Eight of the ten (80.0%) patients with negative ^99m^Tc-MDP scans who switched from watchful waiting to salvage radiotherapy had evidence of extraskeletal metastases on ^99m^Tc-PSMA scan. Two of the ten (20.0%) patients with negative ^99m^Tc-MDP scans who switched from watchful waiting to salvage radiotherapy had evidence of regional skeletal metastases on ^99m^Tc-PSMA scan. Figure 5 illustrates a patient who had planned to change treatment but was kept on the original regimen because of the PSMA scan results (1/11, 9.1%).

## Discussion

Published studies have demonstrated the superiority of ^68^ Ga-PSMA PET/CT in the detection of PCa lesions. However, PET/CT is not widely available in less developed countries, and far fewer institutions have PET/CT devices than SPECT/CT devices. The limited production of ^68^ Ga from a single elution of ^68^Ge-^68^ Ga generators, combined with the short half-life of ^68^ Ga (88.9% β +, 67.71 min), results in the need for multiple rounds of production per day to maintain patient use, limiting the number of patient tests per day. With the introduction of PSMA-directed radioligand therapy (RLT) in PCa patients in recent years, interest in this field has increased. Targeted therapy can be effective only if the target protein is adequately expressed in most tumour lesions. Therefore, patients scheduled for ^177^Lu-PSMA RLT should be screened by PSMA preimaging. Nevertheless, preimaging does not require the same high resolution as PET. ^99m^Tc, available from ^99^Mo-^99m^Tc generators, is a nuclide routinely used in SPECT imaging and has good physical properties and is inexpensive and widely available. Thus, ^99m^Tc-based PSMA ligands are a cost-effective clinical alternative to ^68^ Ga products. This study differs from previous reports of ^68^ Ga-PSMA PET/CT and ^99m^Tc-MDP SPECT/CT in that PET/CT is an imaging mode with higher inherent resolution and better signal-to-noise ratio than SPECT/CT, with predictable advantages. In this study, ^99m^Tc-PSMA SPECT/CT and ^99m^Tc-MDP SPECT/CT were systematically compared using the SPECT/CT imaging mode. Few studies have compared ^99m^Tc-PSMA SPECT/CT and ^99m^Tc-MDP SPECT/CT in the detection of PCa bone metastases^11,12^; only one prospective study, reported by Kabunda et al. and including 41 patients, has been published^12^. In contrast to previously reported studies, our study was not only a head-to-head prospective study but also analysed the relationship of bone metastasis size and serum PSA level on the detection rate of bone metastases in the two imaging modes.

### On per-patient analysis

Our results revealed a sensitivity and specificity of 80.0% and 100% for ^99m^Tc-PSMA SPECT/CT and 72.0% and 81.6% for ^99m^Tc-MDP SPECT/CT, respectively. ^99m^Tc-PSMA SPECT/CT was superior to ^99m^Tc-MDP SPECT/CT in the determination of bone regions with metastases. This is probably because the progression of PCa bone metastases begins with red bone marrow seeding, followed by osteoclastic activation and osteoblastic activation^13^. ^99m^Tc-MDP scans will not detect bone marrow metastasis and are less sensitive to dissolved bone lesions and early sclerosing lesions. These data are consistent with previously published data, although the latter were derived from multimodal comparisons of ^99m^Tc-MDP SPECT/CT and ^68^ Ga-PSMA PET/CT. In a report by Pyka et al., the sensitivity was 98.7–100.0% (PSMA) and 86.7–89.3% (MDP), and the specificity was 88.2–100.0% (PSMA) and 60.8–96.11% (MDP)^7^.

### On lesion‐based analysis

The proportion of “typical metastasis” versus “equivocal metastasis” detected by the two imaging methods was 26.3:1 (PSMA) and 2.9:1 (MDP). The number of equivocal lesions was significantly lower in the ^99m^Tc-PSMA scan, helping to reduce additional examinations (such as MRI, PET/CT, PET/MR, etc.), which are often necessary to clarify unclear findings on ^99m^Tc-MDP scans. Thus, the widespread use of ^99m^Tc-PSMA scan may be beneficial for reducing additional examinations and the financial burden on patients.

In recent years, there has been increased interest in treatment decisions for oligometastatic PCa^14^ Oligometastasis is a transitional stage between localized disease and widespread metastasis, with a limited number of metastases and specificity of metastatic organs. Singh et al. reported that patients with PCa with ≤ 5 metastases had similar survival outcomes to those with no metastases, but their prognoses was significantly better than that of patients with > 5 metastases^15^.

This study further compared the differences in the number of bone metastatic lesions detected by the two imaging methods, and the results showed that ^99m^Tc-PSMA was superior to ^99m^Tc-MDP in the detection of small lesions (maximum diameter of 0.6 cm or less). According to the results of this study, the reason for this phenomenon may be that the uptake of ^99m^Tc-PSMA in small lesions is higher than that of ^99m^Tc-MDP. This result is similar to that of a previously published study on ^68^ Ga-PSMA PET/CT and ^99m^Tc-MDP SPECT/CT^16^.

### Imaging and serum biomarkers

PSA levels can better describe the characteristics of PCa and have been included in PCa risk management systems^17^^,^^18^. In the current study, PSA was the main predictor of bone metastatic lesions, with an optimal cut-off value of 2.635 ng/mL for ^99m^Tc-PSMA and 15.275 ng/mL for ^99m^Tc-MDP. Thus, among patients with low PSA levels, choosing the ^99m^Tc-PSMA scan seems to be of greater benefit than choosing the ^99m^Tc-MDP scan.

### Imaging and subsequent therapy modality

**Figure 6:**
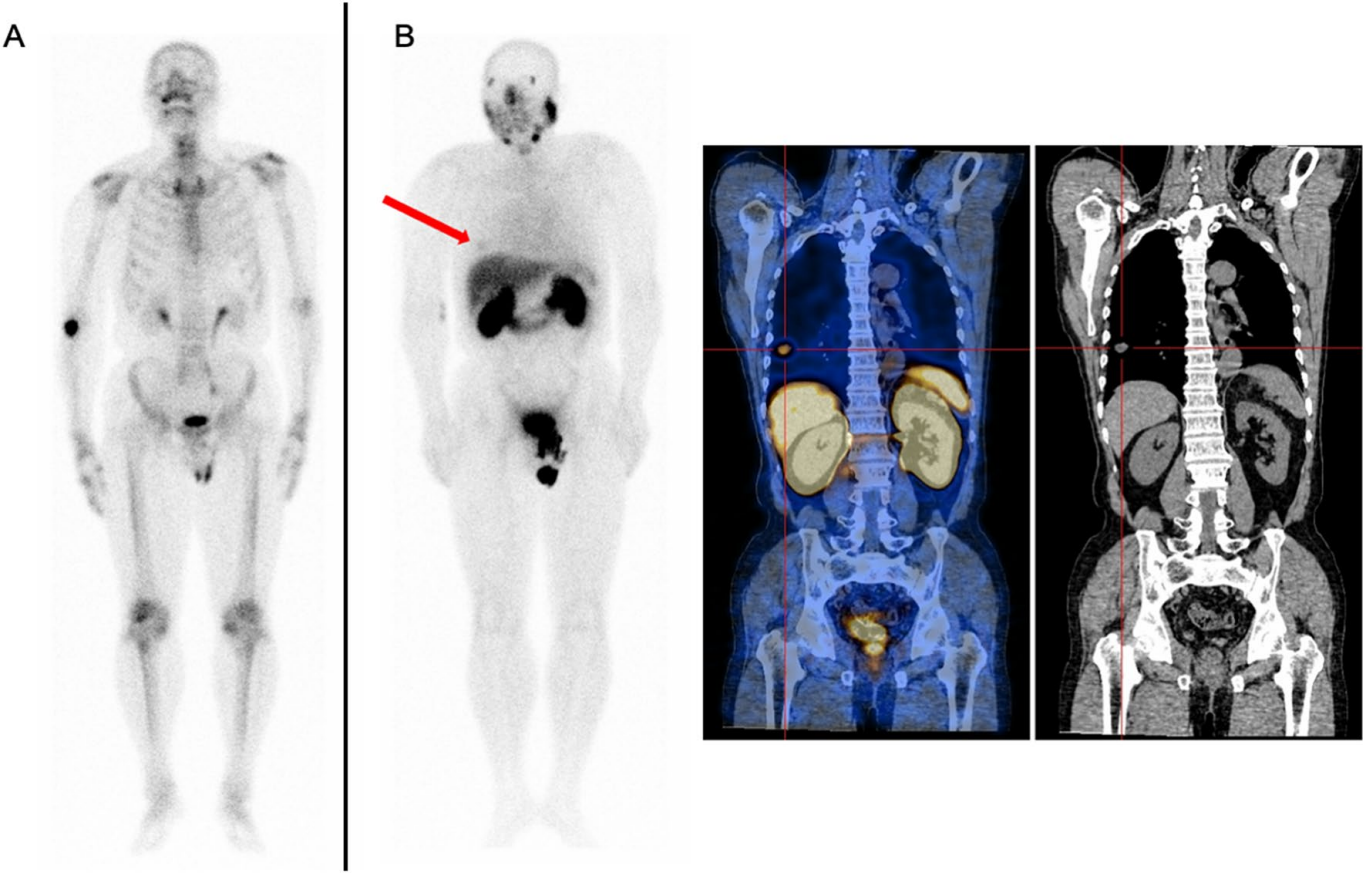
A 66-year-old patient with a BR of PCa. The Gleason score was 7, and the PSA level was 3.14 ng/mL. (**A**): Whole-body planar ^99m^Tc-MDP. (**B**): Whole-body planar ^99m^Tc-PSMA and coronal SPECT/CT. ^99m^Tc-PSMA SPECT/CT helped identify lung metastasis (**B**, red arrow and cross).

**Figure 7:**
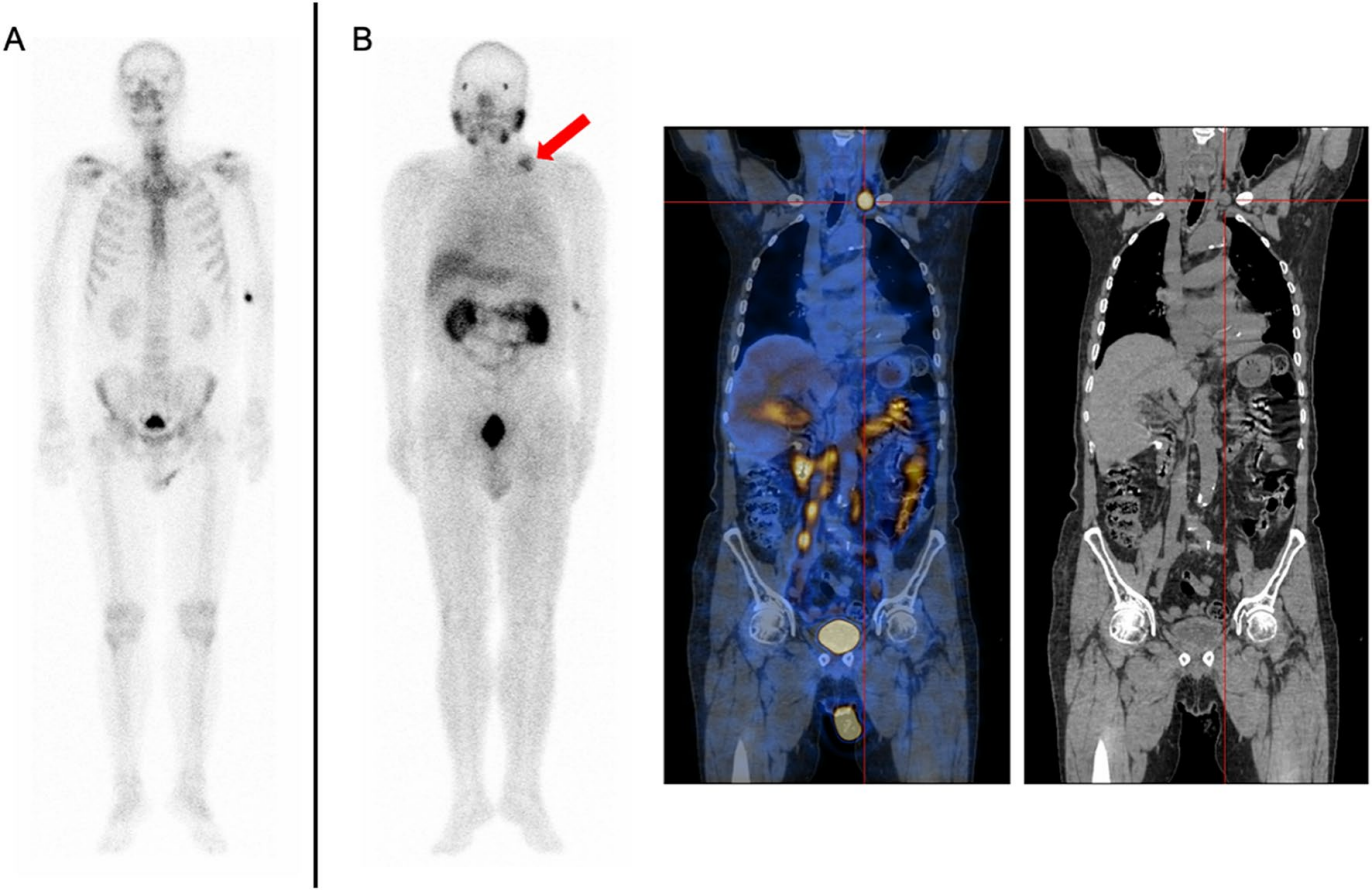
A 67-year-old patient with a restaged PCa. The Gleason score was 10, and the PSA level was 0.11 ng/mL; (**A**): Whole-body planar ^99m^Tc-MDP. (**B**): Whole-body planar ^99m^Tc-PSMA and coronal SPECT/CT. ^99m^Tc-PSMA SPECT/CT revealed lymph node metastases in the left supraclavicular fossa (B, red arrow and cross).

Although our study focused on the difference between ^99m^Tc-PSMA SPECT/CT and ^99m^Tc-MDP SPECT/CT in the diagnosis of bone metastases in PCa, extraosseous metastases are common in patients with advanced PCa. Eight BR and restaging patients in our study group were found to have extraskeletal metastases on ^99m^Tc-PSMA SPECT/CT, such as lung metastasis (Fig. 6) and lymph node metastasis (Fig. 7). Therefore, in patients with advanced PCa, to address the impact of organ overlap and accurately locate metastases, SPECT/CT fusion imaging covering the whole field during the ^99m^Tc-PSMA scan was necessary. The diagnosis of visceral metastasis is crucial for the prognosis and treatment of PCa patients, and visceral or lymph node metastasis > 3 cm is a contraindication for ^223^RaCl_2_ treatment^19^. If local recurrence and/or metastasis is found, salvage surgery or radiotherapy can be selected. Figure 5 illustrates a patient after treatment; his ^99m^Tc-MDP scan showed multiple sites of abnormal uptake of imaging agent in bones throughout the whole body, significantly more than the ^99m^Tc-PSMA scan, indicating high inconsistency between the two examination methods. The reasons for this may be as follows. First, the patients' original bone metastatic tumour burden was reduced after treatment, showing weak or no PSMA expression, which was consistent with the changing trend of serum PSA. Second, the patient may have also been taking zoledronic acid during androgen deprivation therapy (ADT) to repair bone damaged by the metastatic lesions^20^. The high uptake of imaging agents in some bone metastatic lesions on ^99m^Tc-MDP scans may be due to the bone repair effect of zoledronic acid. In conclusion, PSMA scans are more specific than MDP scans in the interim evaluation of the systemic tumour burden status of PCa patients after treatment and are less susceptible to interference with other osteogenic drugs.

In the ^99m^Tc-PSMA scans negative and ^99m^Tc-MDP scans positive group, 5 patients with negative ^99m^Tc-PSMA scans but positive ^99m^Tc-MDP scans were diagnosed with PCa bone metastases during follow-up. This may be due to presenting with a tumour phenotype with insufficient PSMA expression. This finding is similar to that of a previously published study by Afshar-Oromieh A et al.^21^. Sufficient expression of PSMA is an indication for PSMA-RLT, while insufficient expression of PSMA is a contraindication of PSMA-RLT. Patients with reduced PSMA uptake but increased MDP uptake may benefit from radioligand palliative bone therapy^22^. In these cases, a therapeutic regimen using radionuclides such as ^89^SrCl_2_ or ^223^RaCl_2_ may be preferable. Therefore, in some cases, the two imaging methods are not completely substituted for each other.

### Limitations and prospects

Admittedly, this study had some limitations. For ethical reasons, all bone metastases were diagnosed clinically. Due to the small sample size, this study did not conduct a series of studies on the accuracy of different treatment methods in the diagnosis of bone metastasis. Since our patients included primary staging, BR and restaging, the studied population was heterogeneous. Patients with PCa may demonstrate the so-called “flare phenomenon” on ^99m^Tc-MDP scans during ADT treatment (Fig. 5). This phenomenon can lead to false positive results for metastases that undergo osteoblastic healing processes after therapy. Therefore, in the next stage, we plan to investigate the effects of different treatment responses on the detection efficiency of ^99m^Tc-PSMA SPECT/CT and ^99m^Tc-MDP SPECT/CT.

## Conclusion

In this prospective study, our results revealed that ^99m^Tc-PSMA SPECT/CT had higher sensitivity and specificity than ^99m^Tc-MDP SPECT/CT in terms of bone metastasis in PCa. ^99^Tc^m^-PSMA scans clearly diagnosed the nature of the bone lesions, helping to reduce equivocal diagnoses, and demonstrated an additional benefit of providing information on extraskeletal metastases. ^99m^Tc-PSMA is superior to ^99m^Tc-MDP in the detection of small bone lesions and low PSA levels. With regard to therapy, ^99m^Tc-PSMA scan might have utility in further improving the details of the patient’s therapy modality. In brief, ^99m^Tc-PSMA SPECT/CT could have wide application prospects.

## Data Availability

The datasets used and/or analysed during the current study available from the corresponding author on reasonable request.
